# Supplementary figures and images for: Nurses’, patients’, and informal caregivers’ attitudes toward aggression in psychiatric hospitals: A comparative survey study
Source: PLoS One. 2022 Sep 29;17(9):e0274536. doi: 10.1371/journal.pone.0274536 (PMC9522285; doi:10.1371/journal.pone.0274536)

**S1 Table. Comparison of nurses’, patients’, and informal caregivers’ perceptions of aggression**

**
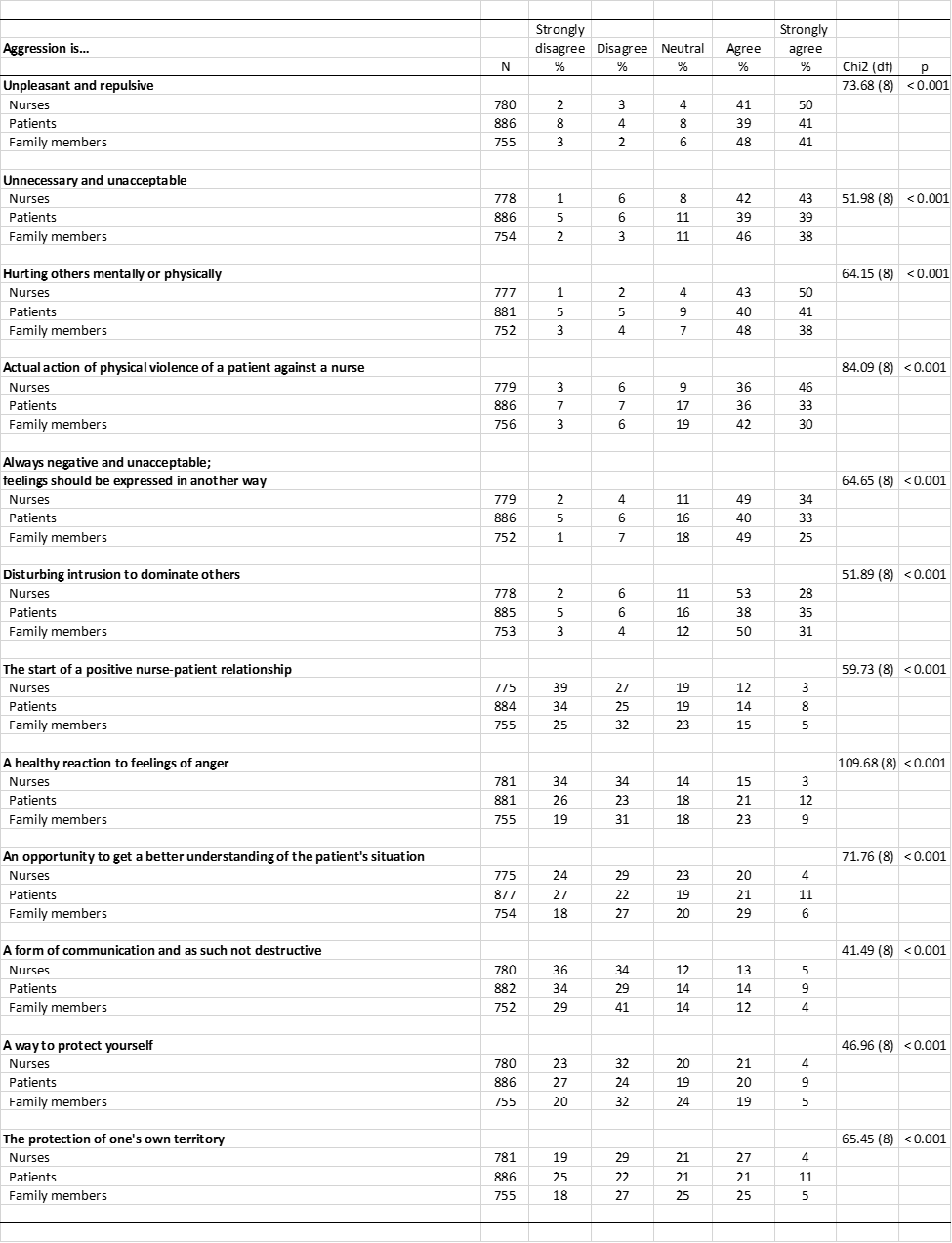
**

Supplement: S1 Table — (DOCX) [file pone.0274536.s001.docx]

**S2 Table. Results of regression analysis for the total sample**

**
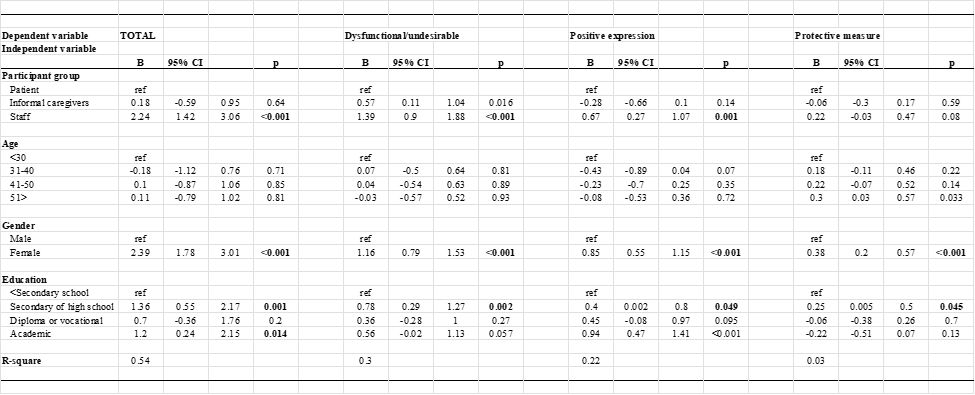
**

Supplement: S2 Table — (DOCX) [file pone.0274536.s002.docx]

**S3 Table. The results of the regression analysis separately for each participant group**

**
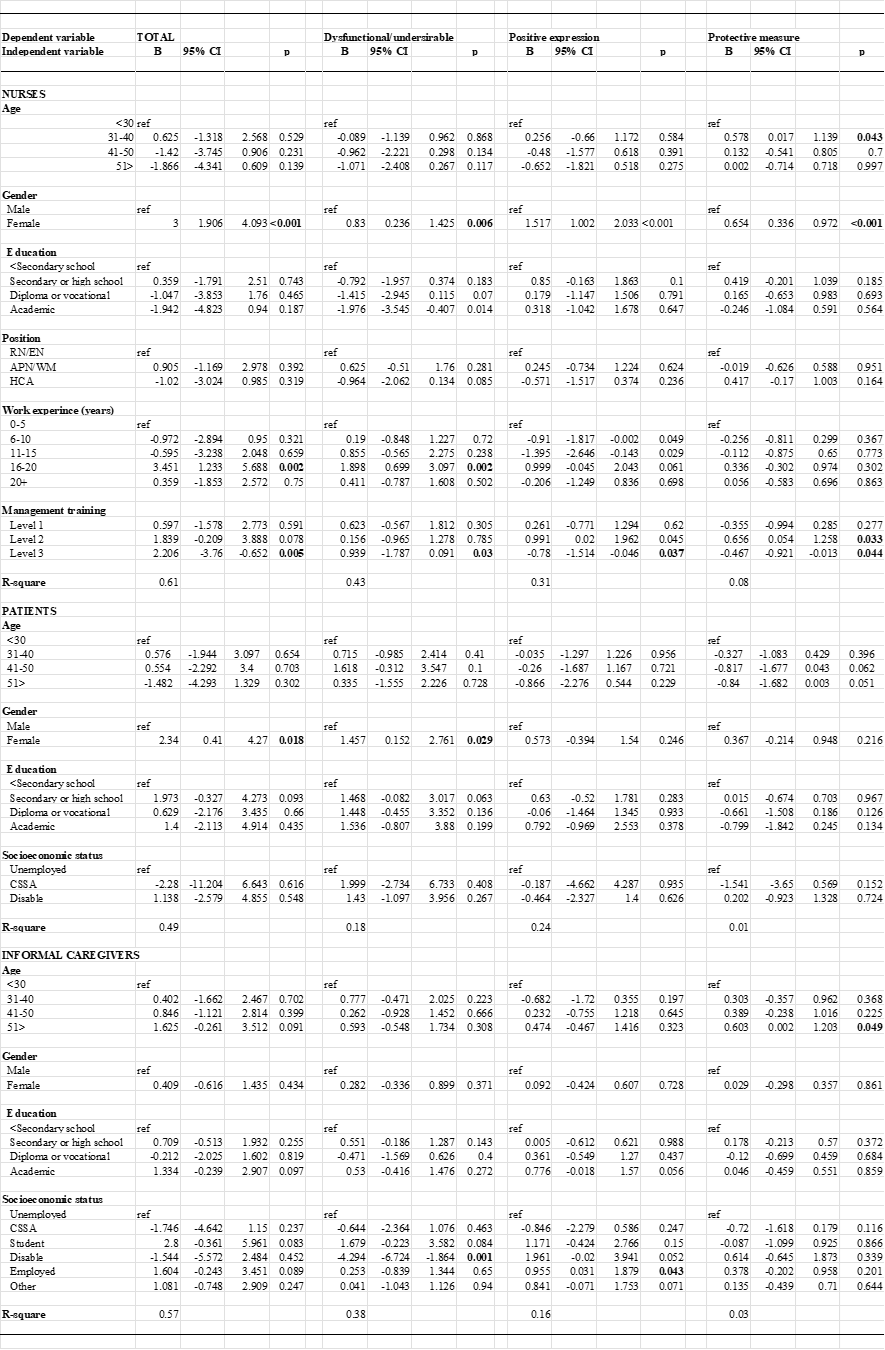
**

Supplement: S3 Table — (DOCX) [file pone.0274536.s003.docx]
