# Supplementary material for: Nurses’, patients’, and informal caregivers’ attitudes toward aggression in psychiatric hospitals: A comparative survey study
Source: PLoS One. 2022 Sep 29;17(9):e0274536. doi: 10.1371/journal.pone.0274536 (PMC9522285; doi:10.1371/journal.pone.0274536)
Supplement: S4 Table — (DOCX) [file pone.0274536.s004.docx]

**S4 Table. Sensitivity analysis to identify possible multicollinearity in the regression model**
