# Supplementary material for: Nurses’, patients’, and informal caregivers’ attitudes toward aggression in psychiatric hospitals: A comparative survey study
Source: PLoS One. 2022 Sep 29;17(9):e0274536. doi: 10.1371/journal.pone.0274536 (PMC9522285; doi:10.1371/journal.pone.0274536)
Supplement: Attachment — Submitted filename: Response to reviewers_050822.docx [file pone.0274536.s005.docx]

Joseph Donlan

Editorial Office

PLOS ONE	August 5, 2022

PONE-D-21-29009

Nurses’, patients’, and informal caregivers’ attitudes toward aggression in psychiatric hospitals: a comparative survey study

Dear Editor,

Thank you very much for your feedback and considering our paper “Nurses’, patients’, and informal caregivers’ attitudes toward aggression in psychiatric hospitals: a comparative survey study.” Please, find in this rebuttal letter a detailed response to each point raised based on the review process as follow:

**Please include additional information regarding the survey or questionnaire used in the study and ensure that you have provided sufficient details that others could replicate the analyses. For instance, if you developed a questionnaire as part of this study and it is not under a copyright more restrictive than CC-BY, please include a copy, in both the original language and English, as Supporting Information.**

**RESPONSE:**

We used the POAS instrument that is under copyright and not able to be included.

**Thank you for stating the following in the Acknowledgments Section of your manuscript:**

**“The research team would like to express deep gratitude to the following people who contributed to this project:** **Ms LF Wong and Mr MH Chow for their support and assistance in this project; and our hospital partners and staff, for their help in facilitating the data collection. Special thanks to all the participants who generously shared their valuable time and experience for the purposes of this project. MV has received funding for this study: the Start-up funding by the Hong Kong Polytechnic University (grant name: Developing user-centered treatment culture to prevent patient aggressive events in psychiatric hospitals, https://www.polyu.edu.hk/en/) and the Academy of Finland fund (grant numbers: 294298, 307367, https://www.aka.fi/en/). The funders had no role in study design, data collection and analysis, decision to publish, or preparation of the manuscript.”**

**We note that you have provided funding information that is not currently declared in your Funding Statement. However, funding information should not appear in the Acknowledgments section or other areas of your manuscript. We will only publish funding information present in the Funding Statement section of the online submission form.**

**Please remove any funding-related text from the manuscript and let us know how you would like to update your Funding Statement. Currently, your Funding Statement reads as follows:**

**“MV has received funding for this study: the Start-up funding by the Hong Kong Polytechnic University (grant name: Developing user-centered treatment culture to prevent patient aggressive events in psychiatric hospitals, https://www.polyu.edu.hk/en/) and the Academy of Finland fund (grant numbers: 294298, 307367, https://www.aka.fi/en/). The funders had no role in study design, data collection and analysis, decision to publish, or preparation of the manuscript.”**

**Please include your amended statements within your cover letter; we will change the online submission form on your behalf.**

**RESPONSE:** The statements have been modified as follows:

**The Acknowledgement:**

Ms LF Wong and Mr MH Chow for their support and assistance in this project; and our hospital partners and staff, for their help in facilitating the data collection. Special thanks to all the participants who generously shared their valuable time and experience for the purposes of this project.

**Funding Statement**

Prof Dr. Maritta Välimäki has received funding for this study (Start-up funding by the Hong Kong Polytechnic University, 1-ZE84, https://www.polyu.edu.hk/sn/research/research-projects/ and the Academy of Finland fund (grant numbers: 294298, 307367, https://www.aka.fi/en/]). The funders had no role in study design, data collection and analysis, decision to publish, or preparation of the manuscript.

**4. In your Data Availability statement, you have not specified where the minimal data set underlying the results described in your manuscript can be found. PLOS defines a study's minimal data set as the underlying data used to reach the conclusions drawn in the manuscript and any additional data required to replicate the reported study findings in their entirety. All PLOS journals require that the minimal data set be made fully available. For more information about our data policy, please see http://journals.plos.org/plosone/s/data-availability.**

**Upon re-submitting your revised manuscript, please upload your study’s minimal underlying data set as either Supporting Information files or to a stable, public repository and include the relevant URLs, DOIs, or accession numbers within your revised cover letter. For a list of acceptable repositories, please see http://journals.plos.org/plosone/s/data-availability#loc-recommended-repositories. Any potentially identifying patient information must be fully anonymized.**

**Important: If there are ethical or legal restrictions to sharing your data publicly, please explain these restrictions in detail. Please see our guidelines for more information on what we consider unacceptable restrictions to publicly sharing data: http://journals.plos.org/plosone/s/data-availability#loc-unacceptable-data-access-restrictions. Note that it is not acceptable for the authors to be the sole named individuals responsible for ensuring data access.**

**We will update your Data Availability statement to reflect the information you provide in your cover letter.**

**RESPONSE**: Thank you for your explanation of the Data Availability Statement. We have shared the basic information of the study and the study dataset at dx.doi.org/10.17504/protocols.io.j8nlkk62wl5r/v1.

**5. We note that you have indicated that data from this study are available upon request. PLOS only allows data to be available upon request if there are legal or ethical restrictions on sharing data publicly. For more information on unacceptable data access restrictions, please see http://journals.plos.org/plosone/s/data-availability#loc-unacceptable-data-access-restrictions.**

**In your revised cover letter, please address the following prompts:**

**a) If there are ethical or legal restrictions on sharing a de-identified data set, please explain them in detail (e.g., data contain potentially sensitive information, data are owned by a third-party organization, etc.) and who has imposed them (e.g., an ethics committee). Please also provide contact information for a data access committee, ethics committee, or other institutional body to which data requests may be sent.**

**b) If there are no restrictions, please upload the minimal anonymized data set necessary to replicate your study findings as either Supporting Information files or to a stable, public repository and provide us with the relevant URLs, DOIs, or accession numbers. For a list of acceptable repositories, please see http://journals.plos.org/plosone/s/data-availability#loc-recommended-repositories.**

**We will update your Data Availability statement on your behalf to reflect the information you provide.**

**RESPONSE:** We have shared the basic information of the study and the study dataset at dx.doi.org/10.17504/protocols.io.j8nlkk62wl5r/v1.

**6. Please amend your list of authors on the manuscript to ensure that each author is linked to an affiliation. Authors’ affiliations should reflect the institution where the work was done (if authors moved subsequently, you can also list the new affiliation stating “current affiliation:….” as necessary).**

**RESPONSE**: We have modified and updated the author/affiliation list accordingly.

**Reviewers' comments:**

Reviewer #1: Thank you for the opportunity to review. Aggression is a major and important issue in mental health care. I agreed with the authors’ perspective to understand patients’ aggression from multiple angles but I thought it is necessary to modify or add some descriptions.

**Abstract**

**Do authors present the results of the regression analysis or logistic analysis? I think it is better to clarify what analysis was based on the results and describe the ORs or beta including 95%CIs.**

**RESPONSE:** The abstract has been modified accordingly (see below):

Nurses had significantly more negative and less tolerate attitudes (mean (SD)=47.1 (7.5), p<0.001) comparing to patients (mean (SD)=44.4 (8.2)) and informal caregivers (mean (SD)=45.0 (6.9)) regarding a POAS-S total score, and dysfunction and function sub-scores (mean (SD)=25.3 (4.1) and 15.0 (3.6), respectively, both ps <0.001) comparing to patients (mean (SD)=23.7 (5.3) and 14.0 (4.1), respectively) and informal caregivers (mean (SD)=24.4 (4.2) and 13.9 (3.5), respectively).

**BACKGROUND**

**Authors said, ‘attitudes towards aggression plays a crucial role in how aggressive incidents are seen, occur or are managed in health care settings.’ However, the relationships between attitude toward aggression and other factors are not clear. I think further explanation about the relationships is needed.**

**RESPONSE:** The following text has been added to the BACKGROUND (in pages 4-5) section to explain the relationship between attitudes toward aggression and their impact on clinical practice:

On the other hand, aggression is a complex process, which integrates social and cognitive aspects of those persons who are involved, affected by a combination of their personality, individual traits, situation, and decision-making processes.^14^ Attitudes toward aggression plays a crucial role in how aggressive incidents occur, are seen, and are managed in healthcare settings.^15^ Weltens et al.^16^ described that development and expression of aggression is a multifactorial event, which includes attitudes toward patients, cultural factors, and clinician-related factors such as attitudes toward aggression. McCann et al.^17^ concluded, based on their study, that attitudes toward the management of aggression are complex and contradictory: staff’s attitudes can affect the way they manage aggression, and therefore, a wide range of initiatives are needed to prevent and deal with patient challenging behavior. Recent studies have also described the association between attitudes and practices. Efkemann et al.,^18^ for example, confirmed, using a vignette study, that a general approving attitude toward coercion significantly influenced decisions around coercion in individual cases and resulted in a more likely approval of applying coercion.

**In addition, what is the definition of attitude? In the scale name of POAS, it is used the word ‘perception’. It seems that they are different concepts. Would you tell me the reasons that it is appropriate to use ‘attitude’ as perception?**

**RESPONSE**: Thank you for raising this very important point. We did a search using POAS as a keyword to identify how researchers have referred to the key concepts of ‘perceptions’ and ‘attitudes’. We made the following conclusion.

Originally, **the concept of ‘perception’** was referred to in relation to aggression (Jansen, G., Dassen, T., & Moorer, P. (1997). The perception of aggression. Scandinavian Journal of Caring Sciences, 11(1), 51–55. <https://doi.org/10.1111/j.1471-6712.1997.tb00430.x>).

Later on, authors started using the concept of **perception alone:**

1) BILGIN, TULEK, Z., & OZCAN, N. (2011). Psychometric properties of the Turkish version of the Perception of Aggression Scale. *Journal of Psychiatric and Mental Health Nursing*, *18*(10), 878–883. <https://doi.org/10.1111/j.1365-2850.2011.01742.x>;

2) Abderhalden, Needham, I., Friedli, T. K., Poelmans, J., & Dassen, T. (2002). Perception of aggression among psychiatric nurses in Switzerland: Perception of aggression. *Acta Psychiatrica Scandinavica*, *106*, 110–117. <https://doi.org/10.1034/j.1600-0447.106.s412.24.x>;

3) Pazvantoglu, Gumus, K., Boke, O., Yildiz, I., & Sahin, A. R. (2011). Perception of patient aggression among nurses working in a university hospital in Turkey. *International Journal of Nursing Practice*, *17*(5), 495–501. <https://doi.org/10.1111/j.1440-172X.2011.01967.x>;

4) Keser Özcan, Bilgin, H., Akın, M., & Badırgalı Boyacıoğlu, N. E. (2015). Nurses’ attitudes towards professional containment methods used in psychiatric wards and perceptions of aggression in Turkey. *Journal of Clinical Nursing*, *24*(19-20), 2881–2889. <https://doi.org/10.1111/jocn.12903>; Muro, Tomas, C., Moreno, L., & Rubio, V. (2002). Perception of aggression, personality disorders and psychiatric morbidity in nursing students. *Acta Psychiatrica Scandinavica*, *106*(s412), 118–120. <https://doi.org/10.1034/j.1600-0447.106.s412.25.x>).

Other authors have also used the concepts **perception and aggression at the same time:**

1) DE BENEDICTIS, DUMAIS, A., STAFFORD, M.-C., CÔTÉ, G., & LESAGE, A. (2012). Factor analysis of the French version of the shorter 12-item Perception of Aggression Scale (POAS) and of a new modified version of the Overt Aggression Scale (MOAS). *Journal of Psychiatric and Mental Health Nursing*, *19*(10), 875–880. <https://doi.org/10.1111/j.1365-2850.2011.01870.x>;

2) Özcan, Bilgin, H., Badırgalı Boyacıoğlu, N. E., & Kaya, F. (2014). Student nurses’ attitudes towards professional containment methods used in psychiatric wards and perceptions of aggression. *International Journal of Nursing Practice*, *20*(4), 346–352. <https://doi.org/10.1111/ijn.12157>; Ming, Huang, H.-M., Hung, S.-P., Chang, C.-I., Hsu, Y.-S.,

3) Tzeng, Y.-M., Huang, H.-Y., & Hsu, T.-F. (2019). Using Simulation Training to Promote Nurses’ Effective Handling of Workplace Violence: A Quasi-Experimental Study. *International Journal of Environmental Research and Public Health*, *16*(19), 3648–. <https://doi.org/10.3390/ijerph16193648>).

Further, some authors have used **both concepts although referred to the concept of ‘attitudes’ more often (**Palmstierna, & Barredal, E. (2006). Evaluation of the Perception of Aggression Scale (POAS) in Swedish nurses. Nordic Journal of Psychiatry, 60(6), 447–451. <https://doi.org/10.1080/08039480601021803>), while others have mixed the concepts but referred to perceptions (1) Needham et al. (2004). The perception of aggression by nurses: psychometric scale testing and derivation of a short instrument. *Journal of Psychiatric and Mental Health Nursing*, *11*(1), 36–42. <https://doi.org/10.1111/j.1365-2850.2004.00678.x>; 3) Wong, & Chien, W. T. (2016). Testing psychometric properties of a Chinese version of perception of aggression scale. *Asian Journal of Psychiatry*, *25*, 213–217. <https://doi.org/10.1016/j.ajp.2016.10.030>).

In this paper, we refer the concept of attitude using Allport’s (1935) definition: “a mental or neural state of readiness, organized through experience, exerting a directive or dynamic influence on the individual’s response to all objects and situations to which it is related”. Attitude is seen as a mindset or a tendency to act in a particular way due to both an individual’s experience and temperament. Attitudes can also refer to a complex combination of things called personality, beliefs, values, behaviors, and motivations.

**Although authors predicted that nurses would have most negative and less tolerant attitudes toward aggression, what is the basis for the hypothesis? I think it is necessary to add the explanation of why the hypothesis was led with references.**

**RESPONSE**: The evaluator identified a very crucial point in this manuscript, which has now been deleted.

**MATERIALS AND METHODS**

**In Table 1, the number of admissions is shown in a year, but the number of discharged patients is shown in a month. I think it is better to present the periods consistently.**

**RESPONSE:** We deleted the information regarding the number of patients in a month as it may mislead the readers.

**Although the nurse participants group included assistant nurses, is it appropriate to regard assistant nurses as the same members of the professional group? It is considered that the registered and enrolled nurses have the expertise and the experience of the care using them. Do Assistant nurses also have them? If so, the authors have to add an explanation of the nursing qualification system in Hong Kong. Or if not, you have to add the reason including assistant nurses. In addition, is the training for violence management for nurses only, not including assistant nurses?**

**RESPONSE:** In many countries, assistant nurses still form the main healthcare group on the wards although they may have only minimal or hardly any professional training. In Hong Kong, assistant nurses were included because they are the key healthcare workers on psychiatric wards and also manage patient aggressive behavior on a daily basis.

In Hong Kong, assistant nurses participate in the same aggression training (Levels I–III) as those in other nurse professions. In addition, as recommended by the recent Cochrane review by Geoffrion et al. (2020), studies should include other types of healthcare workers, who are also victims of aggression in the same settings, such as orderlies (healthcare assistants) (see the reference by Geoffrion S, Hills DJ, Ross HM, Pich J, Hill AT, Dalsbø TK, Riahi S, Martínez-Jarreta B, Guay S. Education and training for preventing and minimizing workplace aggression directed toward healthcare workers. Cochrane Database of Systematic Reviews 2020, Issue 9. Art. No.: CD011860. DOI: 10.1002/14651858.CD011860.pub2. Accessed 18 July 2022).

Based on the valuable comment, the text in the manuscript (page 11) was modified as follows:

The inclusion criteria for nurses were as follows: registered nurses in psychiatry (Registered Nurse, Psychiatric, RNP), enrolled nurses (Enrolled Nurse, Psychiatric, ENP), and supporting staff without registration or enrolment in the Nursing Council of HK (Health Care Assistant, HCA; and Patient Care Assistant, PCA). All of these groups are potential victims of aggression in the same settings and have regular aggression training at the hospital (Level I: one hour online lecture about the causes, signs, symptoms, and the model of violence, and situation awareness; Level II: half-day workshop about how to disengage when a nurse is attacked; Level III: 1.5 day workshop about control and restraint techniques including team formation, holding skills etc.). Requested by the Occupational Safety Department by the Hospital Authority, Level I training is compulsory for all frontline staff, while Levels II and III are compulsory for those nurses working in psychiatric settings. The Committee of the Hospital Authority has also recommended that Levels I and II should be updated every year. Nursing staff working in psychiatric settings need to complete all three levels in one year. In addition, as the inclusion criteria for nurses, it was assumed that staff were working full time (44 hours/week) at the time of the data collection, and that participation was voluntary.

**The definition of informal caregivers is family members, relatives, and friends. However, I wonder how the relatives and friends did regard as caregivers. If the authors had the other inclusion criteria in this study, it is necessary to add this.**

**RESPONSE:** We defined informal caregivers as follows (page 12):

Informal caregivers, defined as unpaid individuals (a spouse, partner, family member, friend or neighbor) involved in assisting others with activities of daily living and/or medical tasks, were invited to participate in the study.^41^

**The authors need to describe how missing values were handled.**

**RESPONSE:** The following text has been added in the manuscript (page 17):

Pairwise deletion was used to manage missing values (i.e. records without missing data were used in any particular analysis).

**RESULT**

**The length of work experience was categorized into 5 groups in the method section. However, in the result section, it seems to be categorized into 4 groups. Which is the categorization correct?**

**RESPONSE**: Nurses’ work experience is categorized into five categories (Table 6).

**In the nurses' group, there may be a correlation strongly between age and work experience. I think the authors have to explain how the authors considered and analyze the multicollinearity.**

**RESPONSE:** Multicollinearity was managed as follows (page 17):

In addition, to reduce possible multicollinearity in the regression model, we identified the variables that were the most collinear, i.e. nurses’ age and length of work experience. We then conducted a sensitivity analysis by removing the variable ‘work experience’ out of the regression model and then restudied the relationship between the dependent variable (attitude to aggression) using a single independent variable (age) in the regression model.

**I think it is better to add the results about the fitness of the models such as the adjusted R-squared in Table 5 and the result of the Hosmer-Lemeshow test or the Nagelkerke’s R squared in Table 6.**

**RESPONSE:** R-scores have been added in Table 5 and Table 6. The following text has been added to the Analysis section (page 17):

To evaluate the goodness of fit of the logistic regression model and the power of explanation of the model, Nagelkerke’s R squared was calculated.

**Discussion**

**What does the sentence mean, ‘The finding is interesting when compared with those reported previously showing that training could produce at least short-term positive improvements in nurse attitudes towards patient aggression’? Does it mean that the findings present the long-term impacts of training, or these findings did not present the impacts of training on attitude toward aggression? It is necessary to describe the interpretation of these findings clearer.**

**RESPONSE:** The paragraph has been modified as follows (page 27-28):

On the other hand, health care staff in this survey was the only group of respondents who had been trained to manage patients’ aggressive behavior in inpatient psychiatric services. At the time of the data collection a great majority of staff members (88–74%) had participated in aggression management training, which targeted understanding aggression from different perspectives, and offered hands-on skills in how to protect against patient attacks. Nurses who had participated in all three levels of the training (Levels I–III) had still significantly less tolerant attitudes toward patients despite the general clinical assumption that education and training programs for staff reduce or eliminate patient aggressive behavior at work. Some previous studies have still shown that training could produce at least short-term positive improvements in nurses’ attitudes toward patient aggression.^63,64^ However, a recent Cochrane review showed that the evidence is very uncertain about the short-term effects of education and training on aggression, while on the long run, education may not reduce aggression at all, compared to no intervention^.65^ It has also been found that staff training related to the management of patient aggression can be even harmful and cause injuries.^66^ Therefore, the use of training for changing attitudes should be carefully considered as it remains unclear how attitudes could be changed to be more tolerant.^30,48,67^

**It is understandable that female nurses had lower tolerance toward aggression than males. I think it is because of not only the lack of experience and skills but also the differences in biological characteristics. Therefore, I think it may be difficult to suggest the lack of skill simply and it may be also important to build a suitable working environment and support system.**

**RESPONSE:** Based on the literature, we agree that education and training alone are not enough to support healthworkers’ tolerance toward aggression. Therefore, different support systems should be developed. This important point has been added in the Discussion section (page 28-29).

However, we are unsure based on the literature whether female nurses have lower tolerance toward aggression than men.

Females and those with a higher education level in our study had higher total scores and sub-scores regarding dysfunction, function and protection, indicating that females and those with a higher education level had lower tolerance toward aggression than males and those with secondary education and below. This result is consistent with a study reported by Jansen et al.,^15^ which found that female nurses perceived aggression as a destructive phenomenon more commonly than their male colleagues did. In a study conducted in Japan, the authors speculated that female nurses who faced patient aggressive behavior may concentrate on their own distress, which hinders their ability to respond to external attacks.^68^ If patient aggression events are typically handled by male nurses with a lower education level, and more educated nurses are responsible in administrative tasks at the nurses’ station, female nurses’ may lack the experience and skills needed to handle these challenging situations. More research is therefore needed to understand nurse attitudes and management styles of patient aggressive events at the ward level in Asia.

**The nurses’ length of working experience was categorized into five groups. But this categorization seems to make the interpretation of the results difficult. Although the authors described the possible impacts of age, there are no explanations about working experience. How did the author consider the result that only one group with experience of 16-20 years had affected aggression?**

**RESPONSE:** The following text has been added to the Discussion section to discuss possible explanations related to the length of working experience (page 29):

Previous studies have also found negative association between the length of work experience and a positive attitude toward aggression.^19,24^ Verhaeghe et al.^13^ proposed, based on their results, that more experienced nurses may lose a positive perspective and tolerance toward aggression. This development over time toward a tendency to place blame can be explained by the possible impact of patient aggression on nurses. The authors also found that burnout and post-traumatic stress increased significantly for mental health nurses employed more than 10 years. If these assumptions are valid, our finding is crucial from the point of view of clinical practice.
